# Novel Insights into Exogenous Phytohormones: Central Regulators in the Modulation of Physiological, Biochemical, and Molecular Responses in Rice under Metal(loid) Stress

**DOI:** 10.3390/metabo13101036

**Published:** 2023-09-26

**Authors:** Saqib Bilal, Syed Saad Jan, Muhammad Shahid, Sajjad Asaf, Abdul Latif Khan, Lubna, Ahmed Al-Rawahi, In-Jung Lee, Ahmed AL-Harrasi

**Affiliations:** 1 Natural & Medical Sciences Research Center, University of Nizwa, Nizwa 616, Oman; 2 Agriculture Research Institute, Khyber Pakhtunkhwa, Mingora 19130, Pakistan; 3 Department of Engineering Technology, University of Houston, Sugar Land, TX 77479, USA; 4 Department of Applied Bioscience, College of Agriculture and Life Science, Kyungpook National University, Daegu 41566, Republic of Korea

**Keywords:** rice (*Oryza sativa*) tolerance, heavy metals, plant hormone, biochemical processes, transcription factors, oxidative stress, metals uptake

## Abstract

Rice (*Oryza sativa*) is a research model for monocotyledonous plants. Rice is also one of the major staple foods and the primary crop for more than half of the world’s population. Increasing industrial activities and the use of different fertilizers and pesticides containing heavy metals (HMs) contribute to the contamination of agriculture fields. HM contamination is among the leading causes that affect the health of rice plants by limiting their growth and causing plant death. Phytohormones have a crucial role in stress-coping mechanisms and in determining a range of plant development and growth aspects during heavy metal stress. This review summarizes the role of different exogenous applications of phytohormones including auxin, cytokinin, gibberellins, ethylene, abscisic acid, strigolactones, jasmonates, brassinosteroids, and salicylic acids in rice plants for mitigating heavy metal stress via manipulation of their stress-related physiological and biochemical processes, and alterations of signaling and biosynthesis of genes. Exogenous administration of phytohormones and regulation of endogenous levels by targeting their biosynthesis/signaling machineries is a potential strategy for protecting rice from HM stress. The current review primarily emphasizes the key mechanistic phytohormonal-mediated strategies for reducing the adverse effects of HM toxicity in rice. Herein, we have provided comprehensive evidence for the effective role of exogenous phytohormones in employing defense responses and tolerance in rice to the phytotoxic effects of HM toxicity along with endogenous hormonal crosstalk for modulation of subcellular mechanisms and modification of stress-related signaling pathways, and uptake and translocation of metals. Altogether, this information offers a systematic understanding of how phytohormones modulate a plant’s tolerance to heavy metals and may assist in directing the development of new approaches to strengthen rice plant resistance to HM toxicity.

## 1. Introduction

Rice (*Oryza sativa*) feeds almost half of the global population, making it among the most significant food crops in the world [1]. It is grown in diverse environments including soils that are contaminated with heavy metals (HMs) [2]. Rice plant is significantly adversely affected by the presence of heavy metals causing reduced plant growth [3] and even death [4,5] along with reduced quality and yield of rice grains [6]. HM toxicity, caused by the unintended accumulations of Cadmium (Cd), lead (Pb), and Arsenic (As), is emerging as a major factor affecting the performance and yield of rice plants [7]. Photosynthesis is impaired due to less uptake of Fe in the presence of Cd and Zn in moderate dry soil conditions [8]. A decrease in rice plant leaf number, biomass, and area has been reported in the presence of heavy metal stress [9].

**Figure 1:**
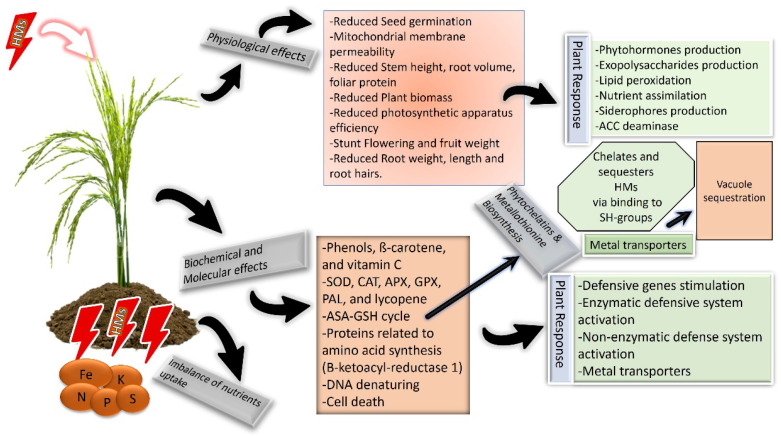
Plant molecular, biochemical, and physiological responses against heavy metals’ direct and indirect toxic effects.

Organic acid contents, amino acids, many sugars, polyols, respiration rate, and glutathione reductase activity increase under Cd stress. The DEGs (Differentially Expressed Genes) of photosynthesis-antenna proteins are downregulated in Cd stress (Table S1 describes the full list of abbreviations) [10]. Heavy-metal-associated proteins (HMPs) are involved in heavy metal transport and detoxification in plant cells and over 46 OsHMPs in rice are identified through in silico analysis [11]. Convincing evidence indicates that the exposure of crops to heavy metal stress increases the levels of some phytohormones including salicylic acid, jasmonate, ethylene, brassinosteroids, and abscisic acid while decreasing other phytohormones such as gibberellins, cytokinin, and auxins [12]. HMs retard the growth, antioxidant activities, chlorophyll content, and photosynthetic rate, and raises the reactive oxygen species (ROS) production in plants [13]; different physiological, biochemical, and molecular responses of rice are illustrated in Figure 1. Whereas application of exogenous phytohormone can modulate plant adaptation responses by regulating the stress expression of stress-related genes and proteins which culminate in HM-induced toxicity. Previous studies have shown that plant hormone-modulated tolerance mechanisms may increase rice’s ability to withstand a variety of abiotic challenges including HM stress [7,14]. Interestingly, there is now a lot of interest in using phytohormones exogenously to control the harmful consequences of HM stress.

**Figure 2:**
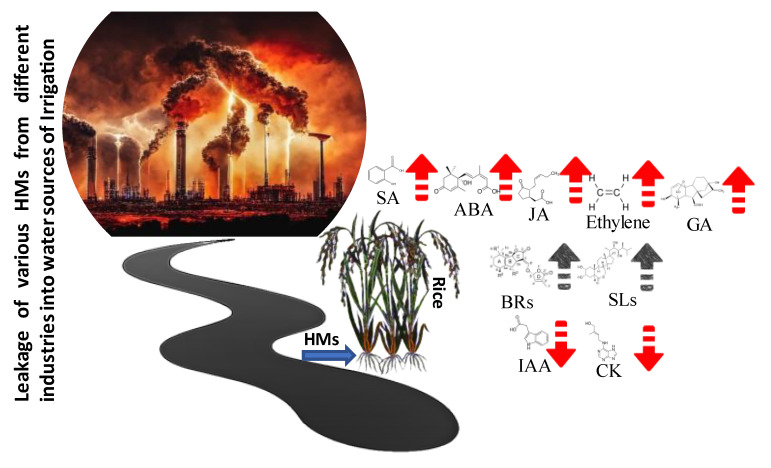
The general trend of phytohormonal response to HM stress in rice. The upward black arrows suggest that there is no direct evidence to support an increase in Brs and SLs during heavy metal stress. ABA, abscisic acid; Sa, salicylic acid; JA, jasmonic acid; GA, gibberellic acid; IAA, indole-3-acetic acid, CK; cytokinin; SLs, strigolactones; BRs, brassinosteroids.

Exogenous application and regulation of endogenous levels of phytohormones through the regulation of their biosynthesis and signaling pathways are promising strategies for protecting rice plants against HM-induced stress (Figure 2). Scientists have used a variety of research techniques, including genomics, gene expression profiling, bioinformatics, microarray, mutant screening, synthetic biology, proteomics, characterization of different biomolecular pathways linked to phytohormone-based HM stress resistance, adaptation, and regulation in plants including rice [15]. Thus, the most recent review in this context primarily concentrated on the major intrinsic advancement in the significant role of phytohormones such as auxin, abscisic acid, gibberellic acid, jasmonate, strigolactones, cytokinins, ethylene, brassinosteroids, and salicylic acid in ameliorating HM-induced toxicity in rice plants. Moreover, various biochemical processes and redox-balancing biomolecular systems involved in counteracting the lethal effects of HM toxicity with special emphasis on augmenting rice plant growth, physiological responses, and uptake and translocation HMs are elucidated. This review may offer a theoretical foundation and direction for reducing HM-induced abiotic stress in rice by employing different convincing strategies to improve agricultural output and ensure food safety.

## 2. Auxin and Signal Transduction

**Figure 3:**
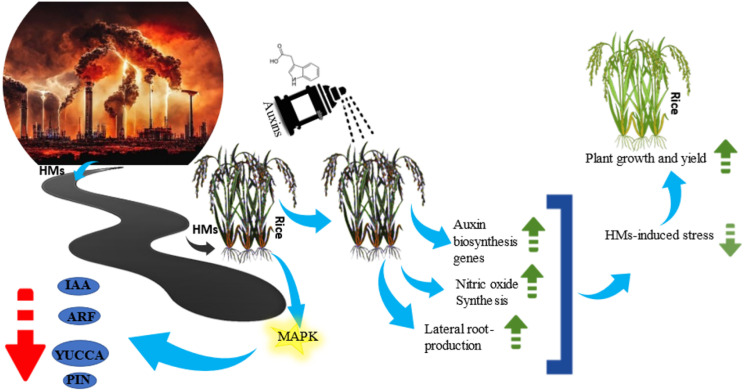
The effect of exogenous auxin application on rice plants under heavy metal stress.

Auxin is among the critical phytohormones required for plant growth and development [16] and is frequently reported as a crucial mediator in various aspects during heavy metal (HM) stress [17]; various attempts at exogenous IAA application for augmented heavy metal stress tolerance in rice are listed in Table 1 and Figure 3. In rice, the primary auxin response genes are stimulated by auxin application [18]. The biochemistry, physiology, and morphology of plants are negatively affected by the elevated concentrations of HMs [17]. Under Cd stress, auxin signaling genes including *IAA*, *ARF*, *PIN*, and *YUCCA* are repressed by mitogen-activated protein kinase (MAPK) in rice [19]. Despite the decrease in the level of endogenous auxins during heavy metal stress, the exogenous treatment of auxins can help maintain the levels of endogenous auxins [17]. However, under normal conditions, the growth of seedlings in both the wild type and transgenic *pmei12* lines were restricted by the high concentrations of NAA (1.0 and 10 μmol L^−1^) [20].

The endogenous auxins degrade during Cd stress in rice, while the application of exogenous tryptophan (L-TRP—auxin precursor) enhances the yield and growth of rice during Cd stress through stabilizing the level of endogenous auxins leading to an increased Cd uptake in the rice straw while the Cd translocation towards rice grains decreases [21]. Selenium is an essential nutrient for rice plants and considered crucial for mitigating oxidative stress, immunity enhancement and activation of stress-related metabolic processes, and endogenous phytohormone modulation under HM stress [29]. Similarly, the treatment of rice with auxin in combination with selenium (Se) reduced the As-induced stress more efficiently than using individual treatment [22]. The co-application of Se and auxin augmented the biomass of rice seedlings, modulated cysteine and proline accumulation, and improved genomic stability by assessing changes in RAPD profile. The treatment of rice seedlings with auxins increases the synthesis of nitric oxide in the roots [23], while during Hg-stressed conditions the use of exogenous nitric oxide induces auxin transport in roots, leading to an improved resistance of rice to Hg-stress but interestingly, during iron deficiency, the auxin levels are decreased by nitric oxide (NO), eventually inhibiting root elongation (Table 1) [24]. In rice, the exogenous application of auxins mitigated the alterations in the root system caused by Cd through increased nitric oxide content and lateral root production [25]. In leaf tissues, photosynthesis is one of the major sources of ROS production. Heavy metal stress alters the rice photosystem II photochemical efficiency. Auxins regulate transcription factor RSL4 through auxin response factors that control ROS production [30]. OsMYB-R1 regulates auxin and salicylic acid crosstalk and the overexpression of *OsMYB-R1* in rice plants exposed to hexavalent chromium (300 µM), and other abiotic stresses resulted in the upregulation of antioxidative genes such as *CAT*, *SOD*, and guaiacol peroxidase [26]. IAA at a concentration of 2.0 μmol L^−1^ reduced As translocation and decreased the As concentration in rice as well as improved plant morphology and biomass [27]. Modulating stress-related signaling pathways by phytohormonal crosstalk in rice plants has been instrumental in strengthening understanding regarding the mechanisms associated with plant responses to hostile environmental conditions. Hormones function in tandem, frequently engaging in synergistic or antagonistic crosstalk under stress conditions. The role of auxin in mitigating HM stress including Cd was postulated from regulation of *IAA*, *ARF*, *PIN*, and *YUCCA* suppression by mitogen-activated protein kinase (MAPK) in rice [19].

Furthermore, the overexpression of auxin biosynthesis rate-limiting enzyme, encoded by the *YUC* gene, leads to enormous propagation of crown roots in rice while *TAA1* (involved in the production of IAA precursor) disruption leads to a reduction in crown root development. The exogenous treatment of auxin could initiate the expression of the *WOX11* gene for the crown root proliferation induced by *YUC* overexpression [31]. These findings revealed that the synthesis of auxin via the TAA/YUC pathway is required and sufficient for rice crown root development. Auxin induces WOX11 transcription, which promotes crown root initiation and development, thus forming the YUC-Auxin-WOX11 module for crown root development in rice. Therefore, the overexpression of *YUC* may also lead to the enhanced tolerance of rice plants toward heavy metal stress.

## 3. Abscisic Acid

**Figure 4:**
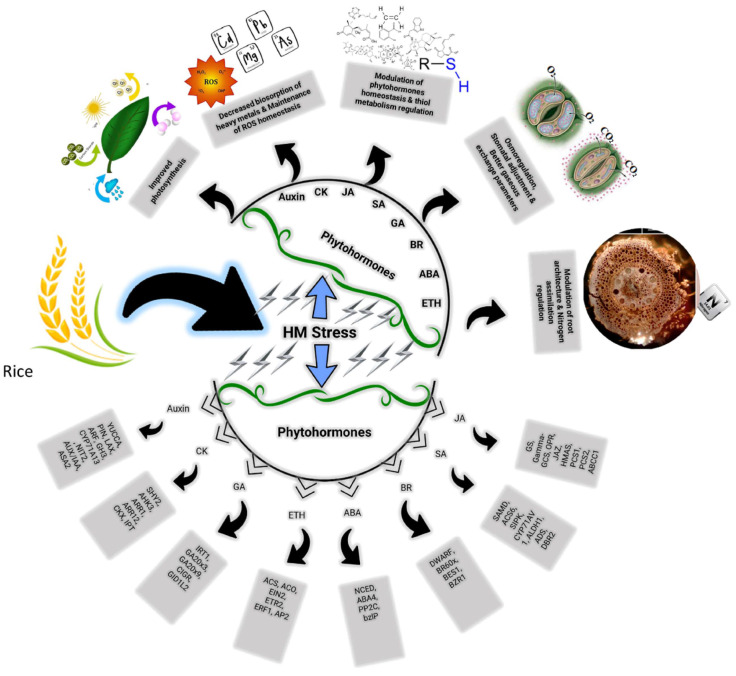
Beneficial effects of phytohormones for development of improved plant tolerance against HM toxicity and induction of a range of regulatory and signaling genes.

Abscisic acid (ABA) is among the first phytohormones responsible for initiating plant resistance to heavy metal toxicity such as Pb, Cd, and As [32]; its diverse roles in rice under HM toxicity are listed in Table 2 and Figure 4. The increase in ABA concentrations after exposure to heavy metals in plants indicates that this phytohormone is involved in the defensive mechanisms of plants towards HM toxicity [17]. Signaling and biosynthesis genes of ABA are upregulated during heavy metal stress [33]. The exposure of plants to As (25 µM) strongly increased the expression of two ABA biosynthesis genes, *OsNCED2* and *OsNCED3*, and upregulated four ABA signaling genes [33]. The rice plants treated with Cd showed an increased level of endogenous ABA [34]. In rice treated with vanadium (V), the gene expression of ABA biosynthesis and signaling genes were triggered in roots [28]. Endogenous levels of ABA increased in rice plants during heavy metal stress [34]. ABA (19 µM) can potentiate the effects of heavy metals by accumulating carbohydrates and inhibiting growth [35]. Heavy metal toxicity increased in rice cultures treated with ABA, leading to a decrease in the storage products’ translocation to sink organs from the source and inhibiting the growth of young leaves. This could be the adaptation strategy of plants to survive in adverse conditions and enable their recovery after the elimination of toxins [17]. Rice plants exposed to Cd showed an increase in endogenous ABA levels, partially because of the ABA biosynthesis gene’s upregulation [32]. During heavy metal exposure, ABA signaling in rice was modulated in plants treated with silicon (Si) leading to a decrease in heavy metal uptake in the roots. The silicon application initially lowered the levels of ABA in plants under heavy metal stress, but when the metal stress periods increased, Si exponentially upregulated ABA biosynthesis. During Si treatment and stress conditions, ABA showed antagonistic behavior with SA/JA biosynthesis [34]. The stress membrane protein (*OsSMP1*) is upregulated in rice plants treated with ABA, and overexpression of *OsSMP1* increases tolerance to heavy metal stresses (CuSO_4_ and CdCl_2_) but increases sensitivity of rice to abscisic acid [36]. Positive correlation between Cd tolerance and endogenous ABA content was indicated [32] upon exposure of Cd-sensitive and Cd-tolerant rice genotypes in which rapid ABA production was detected in roots and leaves of Cd-tolerant rice genotypes compared to Cd-sensitive cultivars [37]. During Cd stress, ABA treatment of up to 100 μM caused stomatal closure, reduced transpiration rate, and dramatically reduced the accumulation of Cd in the leaves of rice plants. *OsNramp5* is the major Cd uptake transporter in rice, hence ABA does not affect Cd absorption [32]. The vesicle membrane protein heavy metal ATPase 3 (HMA3) is considered responsible for the uptake of Cd and Zn [38]. In rice, *OsHMA3* is involved in Cd segregation of Cd into root vesicles [39]. Exposure of As altered phosphatase, H^+^/ATPase, alkaline phosphatase, ROS, and antioxidative and proline biosynthesis genes while the plants pretreated with ABA regulated phosphatase, H^+^/ATPase, and alkaline phosphatase to moderate phosphate, upregulated antioxidative biosynthesis genes and downregulated ROS biosynthesis genes [40]. Similarly, Rice *HMA2* mutants demonstrated decreased Cd and Zn translocation rates from roots to shoots [41].Under Pb (0.25 mM) stress, the pretreatment of plants with ABA at a concentration of 0.1 g m^−3^ restricted Pb translocation from roots to shoots, decreased malondialdehyde and H_2_O_2_ contents in leaves, and alleviated Pb-induced decrease in plant growth and leaf chlorophyll content [42]. The raised ABA concentrations and regulation of ABA-related genes following exposure to heavy metals signify its involvement in the defensive mechanisms of rice plants against metal toxicity. Moreover, the interaction of ABA with other signaling pathways, such as SA/JA biosynthesis and its impact on stress membrane proteins such as *OsSMP1*, *Heavy metal ATPase*, and *OsNramp5* highlight its complex involvement in rice plant responses to heavy metal stress.

## 4. Ethylene

Initiation of ethylene production is among the metabolic responses during heavy metal stress [4]. Production of ethylene depends on the stress duration and intensity such as the concentrations of heavy metals [43]. Table 3 and Figure 4 provide an overview of endeavors focused on manipulating plant physiology by ethylene application to confer increased heavy stress tolerance to rice plants. The initiation of ethylene production to manipulate plant physiology through ethylene application has shown promising impacts in conferring enhanced tolerance to heavy metal stress in rice plants. The use of ethylene biosynthesis and signaling antagonists, cobalt and silver, along with copper oxide nanoparticles (CuO-NP) at a concentration of 450 mg L^−1^, can reduce the extent of ultrastructural and stomatal damage in rice seedlings by controlling the accumulation of reactive oxygen species (ROS) [44]. In rice, ethylene participates in chromium (Cr) stress signaling, as the transcriptomics analysis revealed that the expression of genes related to ethylene biosynthesis such as *ACO5*, *ACO4*, *ACS2*, and *ACS1* were increased in rice treated with chromium, suggesting the involvement of ethylene signaling in rice under metal stress [5]. Moreover, ethylene signaling was induced in response to chromium stress by upregulating *OsEIN3;4* and thereby modulated chromium-induced growth inhibition in rice roots. MAPKs phosphorylates enzymes 1-aminocyclopropane 1-carboxylate synthase ACS6 and ACS2 enzymes to increase the half-life of these enzymes during heavy metal stresses [45]. During Cr stress, ethylene is identified as one of the major signals in rice by activating ACS and ACO gene family members and may contribute to stress mitigation and growth inhibition along with other hormones [46]. The expression of genes related to ethylene biosynthesis such as *ACO5*, *ACO4*, *ACS2*, and *ACS1* were increased in Cr-treated rice plants [17]. The rising water level causes heavy metal contamination in rice and the internal signaling in the plant is mediated by ethylene biosynthesis and signaling, vesicle trafficking, and regulation of ROS, which are the intermediators for gene expression. These ROS and ethylene often prevent the plant’s death during stress [46]. The expression of ethylene biosynthesis genes such as *ACS2* and *ACS6* was induced under ZnO NPs stress and led to enhanced enzymatic antioxidant activities and induced ultrastructural changes. Moreover, mercury and/or Se triggered the abundance of S-adenosylmethionine (SAM), which is involved in the biosynthesis of ethylene and ethylene transduction gene in *Oryza sativa* [47,48]. Ethylene (400 μM) can enhance plant tolerance potential to Cu toxicity by alleviating Cu-induced poisonous effects [49]. Additionally, ethylene responsive factors (*AP2*/*ERF*) overexpression is reported for the activation of phytohormonal crosstalk by accumulation of JA, linolenic acid, ABA and their biosynthesis genes in rice [50]. Moreover, plasma membrane H^+^-ATPases are considered crucial for modulating abiotic stress including heavy metal responses in plants. However, H^+^-ATPase activity was substantially retarded in rice plants treated with ethylene precursor ACC under high pH and led to ethylene-mediated retardation of rice growth, suggesting that ethylene plays an upstream regulator’s role that suppresses H^+^-ATPase activity. The crosstalk between ethylene-responsive factors and various phytohormones, including JA, linolenic acid, and ABA, highlights the complex regulatory network involved in plant responses to heavy metal stress. It is important to note that while ethylene plays a crucial role in stress responses, its overstimulation, as seen in the retardation of H^+^-ATPase activity, can lead to adverse effects on plant growth. The involvement of ethylene in signaling pathways, such as MAPK-mediated phosphorylation of enzymes, modulation of ROS via antioxidant regulation, and upregulation of key genes related to ethylene biosynthesis further underlines its importance in the plant’s adaptive responses to heavy metal stresses.

## 5. Strigolactones

Rice contains three enzymes for biosynthesis of strigolactones (SLs) such as carotenoid cleavage dioxygenase 8, carotenoid cleavage dioxygenase, and β-carotene isomerase encoded by *DWARF 10 (D10)*, *D17* and *D27* [51]. *MAX1*-like genes produce diverse strigolactones in plants via Cytochrome P450 enzymes. *Oryza sativa* has multiple *MAX1* copies, enabling a wide range of strigolactone production with potential implications in heavy metal responses [52]. SLs also play an active role in structuring rhizomicrobiome [53]. Exogenously applied GR24 under phosphate deficiency has been reported to restore normal leaf senescence in SL-deficient mutants [54]. In rice, the uptake and accumulation of As in roots along with the uptake of inorganic phosphate was reduced by SLs [55]. Efforts of exogenous application of strigolactones to improve rice’s ability to tolerate heavy metal stress are summarized in Table 4. SLs play an important role at the cellular level and act as an essential growth regulator by helping rice plants to cope with excessive As toxicity through detoxification mechanisms such as As sequestration via vacuoles and stimulation of cells’ antioxidant defense systems in rice shoots. The SLs trigger other hormonal-responsive pathways and determine plant resilience against stressful environments [13]. Selenium altered *OsSBP1, OsNIP2;1*, *OsPT2*, and low concentration of MJ reduced the gene expression of *OsPt2* and *OsNIP2;1* in roots and *OsSBP1*, *OsCS*, *OsNIP2;1*, and *OsPT2* in shoot to hinder Se uptake [56]. SL mutant rice showed more severe loss of biomass, growth reduction, and phenotypic abnormalities as compared to the wild type Arsenate (As^V^)- treated plants, indicating that SL-deficient d17 and d10 plants are more susceptible to As toxicity. The inferior growth performance of SL-deficient mutants was supported by their reduced photosynthetic pigment contents as compared to the wild type. SL-deficient mutants demonstrate increased susceptibility to As toxicity, highlighting the pivotal role of SLs in mitigating heavy metal stress. The mutants exhibit compromised As detoxification mechanisms, emphasizing the importance of SLs in facilitating As sequestration into vacuoles. In rice shoots, As level was not influenced by SL deficiency; however, the higher vulnerability of SL-deficient plants was due to the compromised As detoxification mechanisms of the cell [56]). Meanwhile, the wild type of rice shoots had higher gene expression of *OsABCC1*, *OsPCS1*, *OsGSH2* and *OsGSH1*, and *GSH*, suggesting higher GSH-assisted sequestration of As into vacuoles [57]. SLs have been reported to restrict the heavy metals’ translocation and toxicity. In rice seedlings, SLs such as (GR24) alleviated adverse effects of Cd and As (As) such as decreased growth. Iron plaque induction with GR24 treatment decreased the content of mineral elements such as Zn, Cu, and Mn while it increased Fe content in rice plants. A potential approach to efficiently inhibit As or Cd uptake and toxicity is by applying GR24 exogenously [58]. During heavy metal stress, the exogenous application of SLs reduces the impact of HM-induced decreased chlorophyll content, photosynthesis, and oxidative stress. It also reduces the lipid peroxidation and ROS level and increases antioxidant enzyme activities that stimulate plant growth. Plant survival can be improved by balancing the phytohormones of plants grown in heavy-metal-contaminated regions. Genes co-expressed with MAX1s (responsible for production of diverse SLs) indicate that Os01g0700900 could have an involvement in rice response mechanisms towards different heavy metal stresses such as copper, chromium, and Cd, while Os01g0701400 expressed in response to As, Os01g0701500 expressed in response to mercury and Cd, Os06g0565100 expressed in response to nickel, mercury As, magnesium, copper and Cd [52]. SLs potentially play a role in alleviating heavy metal stress in plants. Further research is needed to elucidate the specific molecular mechanisms through which SLs mitigate heavy metal stress in plants, including the activation of key genes such as *D10*, *D17*, *D27*, and *OsMAX1*, which promote SL biosynthesis [13]. It has been observed that ABA treatment prevents SL accumulations in the roots of rice seedlings by downregulating *D27* and *D10* expression, which encodes enzymes involved in SL biosynthesis [59]. The multifaceted role of SLs in mitigating heavy metal stress in plants offers promising avenues for further research. Understanding the specific molecular mechanisms underlying SL-mediated responses to heavy metal stress, including their interactions with other phytohormones, holds potential for developing strategies to enhance plant resilience in contaminated environments. 

## 6. Jasmonate

The development and growth of plants under HMs toxicity is stabilized by Jasmonates (JAs), which are lipid-derived naturally occurring hormones. The toxic effects of HMs in plants can be alleviated by JAs through several biochemical and physiological mechanisms and upregulation of genes associated with JAs. The effects of jasmonate application on the heavy metal stress tolerance of rice plants have been compiled in Table 5. The integrity of plant cells is sustained by JAs during HM stress through osmo-protectant biosynthesis and improving antioxidant defense system [60]. JAs improve tolerance and limit accumulation of toxic elements through coordinating chelating capacity, antioxidant enzyme activity, and ion transport channels in plants. JAs may protect crops from metalloids and heavy metals. A role in HM detoxification may be performed by the JA transporters in ATP-Binding Cassette G (ABCG) of the subgroup 4 [61]. In rice, Jasmonates respond to toxic HMs and metalloids through several regulatory mechanisms [62,63,64,65]. Under Antimony (10 to 50 mg L^−1^) stress, the plant responds by significantly increasing methyl jasmonate in rice roots [66]. Rice leaves affected with Cu showed increased level of JAs [67]. Rice growth affected by HMs such as As(III) (Arsenite) was alleviated with exogenous application of MeJAs [65,68]. Pretreatment of rice roots with JA resulted in lower inhibition of root elongation in the presence of As(V) compared to untreated roots [69]. In rice, genes encoding enzymes related to JA biosynthesis such as AOS, 12-Oxo-PDA reductase (OPR), LOXs, and phospholipase are more likely to be enhanced by Cu, causing increased JA accumulation [70]. Furthermore, transcriptomic studies reveal that the roots of rice plants under As(V)-stress have activated the JA signaling and biosynthesis pathways [33], whereas Ref. [71] reported the modulation of the JA signaling pathway in rice plants for overcoming As(III) and As(V)-induced stresses via regulating *LOX*, *OP*, and *JAZ* genes in upland rice shoots. Overall, rice plants treated with Cd and Cu showed higher level of putative *OsAOCs*, *OsAOSs*, *OsLOXs*, and *OsDADs* gene expressions [70,72], while *OsOPRs* are more commonly induced by Cd [72]. The JA production is indicated to be negatively affected by *OsWRKY28*, as the *OsWRKY28* knockout mutant rice plant roots showed upregulation of genes responsible for JA biosynthesis such as *OsJAR1*, *OsOPR1*, *OsAOC*, *OsAOS4*, and *OsLOX1*/*9/11*. The As(III) translocation from roots to shoots was inhibited and the adventitious roots’ number was decreased in the coleoptile photomorphogenesis 2 jasmonate-biosynthetic mutant rice plants under As (III) stress [73]. In different stress conditions such as ozone, wound, pathogen, and HM stresses, the methyl jasmonate (MJ) level drastically increases, and it is considered to be among the most crucial phytohormones that respond to various stresses [74,75,76]. A number of studies have shown the reduction of HMs’ effects through the important role of MJ in several plants [77,78]. MJ improves growth in rice plants under As stress [75]. The yield, biomass production, and chlorophyll contents in rice were increased while the adverse As-toxicity effects were effectively alleviated by MJ application. The cycle of ascorbate-glutathione (AsA-GSH) was regulated and antioxidant activity was increased by MJ in rice under As toxicity. The adaptation of rice varieties to As toxicity was improved by MJ through modulating the As and Fe transporter expression in leaves and roots along with an improved root to shoot translocation of Fe. Rice varieties treated with As showed improved biomass production and growth upon exogenous application of MJ [65]. MJ enhances the yield and growth of rice plants under AS stress by ameliorating oxidative stress with AsA–GSH cycle and increased antioxidant enzyme activity along with modulation of As transporters for reducing As accumulation [65]. Aluminum altered the *ABCC1*, *GSH1*, and *PCS* and MJ upregulated the expression of *ABCC1*, *GSH1*, and *PCS* for Al sequestration in the vacuole [79]. MeJA (0.25 μM) co-application with AsIII (25 μM) resulted in improved antioxidant enzyme activities, chlorophyll content, and biomass than plants treated with AsIII alone. A potential adaptive response to cope with As stress in rice seedlings was shown by the co-application of MeJA with AsIII by modulating the expression of genes involved in AsII detoxification, uptake, JA translocation, and signaling mechanisms [68]. A significant role in plant development and growth under HM stress is played by JA and its methyl esters. It also regulates numerous molecular, biochemical, and physiological mechanisms to reduce the HMs’ detrimental effects. Studies suggest that there may be antagonistic or synergistic cross talks between the signaling pathways of other phytohormones and JAs in plants’ response regulation under HM stress [60]. Application of methyljasmonate to rice plants regulated the transcript accumulation of *PCS1*, *PCS2,* and *ABCC1* and heavy metal ATPases (HMAs) *P1B* under Pb stress and consequently led to the immobilization of Pb in roots and reduced its translocation to shoots, as well as mitigating Pb-induced oxidative stress and modulating chlorophyll metabolism of rice plants [80]. Singh et al., 2014 reported that methyl jasmonate (5 μM) enhanced Cd-tolerance, lowered Cd^2+^ uptake, and improved membrane integrity and ‘switching on’ of the JA-biosynthesis by LOX in rice seedlings under Cd stress [81]. Moreover, the level of JA induced by stress is affected by enhancing the influx of extracellular Ca^2+^ or stimulating H_2_O_2_/NO signaling, both of which are mediated by ABA, illustrating a possible interaction of both hormones. JA also participates in this crosstalk with enhanced Ca^2+^ influx, which ultimately boosts calcium-dependent protein kinase activity and the consequent signaling cascade [82]. The remarkable overexpression of *AtJMT* increases significant levels of MeJA in young panicles of rice plants, resulting to a marked decrease in grain yield and an enhancement in ABA accumulation, which suggests crosstalk between MeJA and ABA [83]. 

## 7. Brassinosteroids

Brassinosteroids (BRs) are a polyhydroxylated steroidal class of plant hormones that help plants to cope with the adverse environmental conditions [84]. The applications of exogenous BRs to augment the resilience of rice against heavy metal-induced hostile conditions are detailed in Table 6. BRs can regulate ion uptake by plant cells, reduce HMs accumulation, decrease the root uptake of metals, increase antioxidant enzyme activity to detoxify the ROS produced by HM toxicity, and enhance the metabolic activity and growth of plants under HM stress [85]. BRs have a role in plants’ responses to a variety of HM stresses. Rice seedlings under chromium stress treated with BRs have demonstrated to reduce increased Cr^2+^ uptake [86,87]. Br application to plants under metal toxicity has been reported to alter antioxidant enzyme activities [87,88,89]. Seed priming with brassinosteroids under chromium stress resulted in altered antioxidative defense-associated genes and upregulated CAT, APX, and POD, while it downregulated the MDA, H_2_O_2_, SOD, and EL to ameliorate Cr toxicity [90]. The exogenous treatment of brassinosteroid in rice under Cr stress upregulated gene expression of glutathione reductase, ascorbate peroxidase (Apx), CAT, and SOD [91]. Br28 and Br24 treatment in rice under Cd and As stress decreased its accumulation and translocation to the rice grains [92] while EBL significantly increased the content of carotenoid by 5.8% and significantly decreased As content in the roots by 32.5% in plants under 5 µM As stress [93]. The interaction of Fe with Br28 and Br24 changed the Cd and As contents to a more reduced level in rice grains, suggesting that the use of Br28 and Br24 in combination with Fe is an effective way of reducing Cd and As contents in grains of rice [94]. Foliar spraying of brassinolide on rice plants under Cd stress resulted in increased root length and root surface area, and CAT, SOD, and POD activities were significantly improved, and the Cd content of rice was decreased by transforming Cd into immobile forms and fixing in the cell wall [95]. The application of Br24 decreased the rice growth but applying IP (iron plaque) in combination with Br24 increased its growth. Xu et al. indicated that the Cd and As accumulation and transport into the shoots of rice may be impeded by the conjunctive effect of IP and Br [96], as content in rice leaves is reduced by Br24L while Cd content is reduced by Br28 without IP. On the other hand, Br28 combined with IP reduced As levels while Br24 combined with IP reduced Cd levels in rice plants, implying that the IP and Br interactions may have important roles in the uptake reduction of Cd and As [96]. The target response of post transcriptional fate may be affected by the multilayer interactions of BRs with other PGRs [97]. BRs and epibrassinolides (EBRs) improve the plant’s antioxidant system and regulates the homeostasis of ions. In rice plants, the treatment of EBRs could decrease the root surface Fe translocation and develop oxidative barriers by modulation of aerenchyma area to alleviate the Fe toxicity’s adverse effects. EBRs are capable of providing tolerance to rice plants under Fe toxicity. Plants under Fe stress treated with EBR increased ROS scavenging, enhanced the activities of enzymes such as peroxidase, ascorbate peroxidase, catalase, and superoxide dismutase, and allowed the plant to cope with Fe toxicity through better oxidative damage mitigation [98]. Plants under Al stress conditions are modulated through antioxidant activity stimulation, and reduction of H_2_O_2_ production and MDA content to better cope with stress by the application of BRs such as 24-EBL. The antioxidant activity-related data were supported by *APX08*, *APX02*, *CATb*, *CATa*, *SOD-Fe,* and *SOD-Cu-Zn* gene expression analysis [99]. However, a significant increase in the roots and shoots concentration of Cd with increased biomass and decreased POD was observed in rice seedlings plants pretreated with BRs [100]. BR signaling pathway interaction with ABA signaling through a set of WRKY TFs under stress condition was explained from the findings of BR signaling defective mutants [101]. The disruption of BR signaling has been reported through ABA-induced remorin protein (OsREM4.1) production in rice which binds to SERK1 and causes inactivation of BR signaling [102].

## 8. Salicylic Acid

Salicylic acid (SA) has a role in a number of plants metabolic and biochemical processes under stress conditions including plants facing HMs stress. This simple phenolic compound helps the plant to counteract HM stress by promoting antioxidant compound stimulation through interacting with other plant hormones such as gibberellin, abscisic acid, and auxins [103]. HM-promoted ROS can be scavenged through the SA-promoted free radicals. The HM-promoted ROS regulates antioxidant enzymes including SOD, APX, and GR activities and the gene expression of various proteins including *OsWRKY45* for protection against Cd toxicity in rice [104]. Experiments demonstrating the utilization of SA to promote the growth and modulate physiological and biochemical responses of rice plants under HM stress are summarized in Table 7. SA pretreatment (0.1 mmol) under Pb stress can increase seedling shoot and root length, improve chlorophyll content, reduce peroxide levels, and alter SOD and APX activities [105] while SA application to culture solution in plants under chromium stress upregulates *OsPCS1*, *OsMT1*, and *OsHMA3* gene expression involved with vacuolar sequestration [106]. Similarly, pretreatment of rice roots with SA improves root growth, reduces ROS levels and membrane damage, and enhances SOD, POD, and CAT activities, as well as GSH contents, and improves non-protein thiols’ concentration in plants under CdCl2 stress [107]. Exogenous SA (100 µM) application alleviated the activity of lipoxygenase (LOX) that caused membrane deterioration under 10 µM HgCl_2_ or 10 µM PbCl_2_. The HM-induced increase in malondialdehyde content, injury index, and leakage of electrolytes was ameliorated by SA. The H_2_O_2_ was decreased by SA in Hg presence while it increased it in Pb presence. SA reversed the increased catalase activity of Pb and the decreased catalase activity of Hg. Thus, the membranes damaged by Pb and Hg were ameliorated by SA [108]. The growth inhibition induced by Cd was considerably relieved by SA treatment and elevated the protein thiol content along with photosynthetic pigment content. The antioxidative enzymatic activities were regulated and ROS such as H_2_O_2_ and O_2_^•−^ accumulation were lowered by SA treatment. In rice treated with Cd, the gene expression of *OsNRAMP2* (involved in metal transport, in particular, irons) was downregulated whereas *OsPCS1* (involved in HM tolerance) and *OsHMA3* (involved in restricting Cd translocation [109]) were upregulated by the application of SA. The tolerance and growth of rice seedlings under Cd stress were positively affected by SA application under pot experimentation [110].

Exogenous application of SA (0.1 mM) through foliar application on rice plants at the mature stage did not show any significant effect on its agronomic traits but increased the leaf’s Cd content and decreased the accumulation of Cd in grains. The SA application through spraying had no obvious impact on other growth stages and other tissues’ Cd contents. The accumulation of Cd was reduced by SA application due to its stimulating effect of Cd fixation and deposition in leaves’ cell walls, hence averting the Cd from being transferred into the rice grains [111]. SA application reduced Cd toxicity in rice leaves by increasing the chlorophyll content and decreasing MDA and H_2_O_2_ accumulation. The accumulation of Cd in rice grains was significantly decreased by the exogenous application of SA through foliar application, which modulated the g expression levels responsible for controlling the accumulation of Cd and Cd translocation. Thus, exogenous application of SA via foliar application on rice seedlings during the vegetative growth period reduced Cd content by decreasing the plant height inhibition caused by Cd and raising the dry shoot weight, and reduction of Cd transportation from leaves to the rice grains [111]. The exogenic SA application restored the plant growth and As(V)-induced oxidative stress while drastically reducing the roots to shoot translocation of As. In shoots, SA also modulated antioxidant enzyme activities and coped with oxidative stress caused by As(V) along with hampering Fe translocation in shoots [112]. The interaction of SA and GA has been reported under stress including HMs and normal conditions in plants. For example, the higher accumulation of SA was experienced by exogenous application of GA_3_, which is related to GA-induced overexpression of the GASA4 gene [113]. Moreover, overexpressed *OsMYB-R1* rice exhibited crosstalk of auxin and SA signaling by differential transcriptional modulation as well as higher auxin and SA accumulation under multiple stress conditions, suggesting the crosstalk of SA and auxin. 

## 9. Gibberellic Acid

Gibberellic acid (GA) is involved in the tolerance of rice toward abiotic stress including heavy metal stress. The application of GA is reported to enhance roots’ elongation in rice, inhibit uptake of Cd content, and reduce lipid peroxidation and nitric oxide accumulation [114]. The impacts of GA on rice growth under HMs stress are summarized in Table 8. GA is involved in the accumulation, sensitivity, and resistance of plants towards heavy metals. GA alleviates Cd toxicity by reducing oxidative stress and NO accumulation and inhibition of Cd translocation. This is aided by the reduced absorption of hemicellulose in the cell wall along with vacuole compartmentation to enhance Cd detoxification. The Cd is retained in the intracellular vacuoles and absorption of Cd is decreased by GA which increases the gene expression of *OsHMA3* and decreases the gene expression of *OsCd1* and *OsNRANMP5* [114]. Moya et al., 1995 reported that the addition of gibberellins to the rice seedlings under HM stress partially reversed the adverse effects of HMs and stimulated the carbohydrate reserve mobilization to seedlings from the seed and enhanced its growth while GA_3_ co-application with ABA inhibited the storage product translocation and thereby impeded the growth [35], while the adverse effects of the metals on the nutrient acquisition were not alleviated by the addition of GA_3_ [115]. The exogenous application of GA_3_ reduced the Fe plaque but raised the Mn plaque. GA_3_ application enhanced the uptake of Mn or Fe and a synergistic effect between the production of Fe plaque and GA_3_ application was detected [116]. The genes *CIGR*, *GID1L2*, *GA20ox*, and *GA2ox* are involved in the modulation of GA signaling genes in response to As stress which might evoke the GA-responsive defense pathways in rice [71]. The *GA2ox3* and *GA2ox9* are upregulated in response to stress which probably enhanced the GA levels [33]. GA signaling and biosynthesis has been shown to intiate crosstalk with other phytohormones for amelioration of the effects of HM toxicity in rice plants. Reduced expression of GAST gene family member (*OsGSR1*) illustrated the interaction between GA and BR in RNAi rice mutants by exhibiting deteriorated phenotypes similar to plants deficient in BR, indicating the interaction between BR and GA [117]. Exogenous GA application triggers gene expression, which is involved in SA biosynthesis and signaling [118]. Moreover, application of GA3 has been reported for reduced accumulation of stress-induced ethylene for overcoming photosynthetic inhibition by Cd toxicity [119]. Moreover, in response to As stress, ethylene responsive *LOX* and *ACO7* genes were downregulated and *GA20ox* and *GA2ox* was significantly upregulated, illustrating the mutual adjustment between GA and ethylene [68]. 

## 10. Cytokinin

Cytokinin (CK) signaling, and biosynthesis-related genes are downregulated and the CK inactivation genes are upregulated and thus may be involved in the rice root growth inhibition during heavy metal stress. The effects of CK on rice under HMs stress are outlined in Table 9. Trans-zeatin (Cytokinin) 20 to 40 μmol L^− 1^ solutions treatments increased plant height and stem width, 1-pyrroline, methylglyoxal, proline, and P5C contents, enhanced *P5CS* and *OAT* activities, and reduced glutamic acid contents. Enhanced activities of enzymes involved in proline metabolism (*ProDH*, *P5CS2*, and *DAO4*) expression upregulated, highlighting an adaptive response to stress [120]. In rice roots, As(V) exposure significantly increased genes related to CK inhibition such as *OsCKX5* and *OsCKX4* while lowering the gene expression of the biosynthesis gene of CK, i.e., *OsIPT4* [33]. Under As(V) stress, the CK signaling genes such as *OsRR20* and *OsHKL1*/*OsCRL4* were also downregulated [33]. The application of kinetin solution on rice seedlings was shown to improve the uptake of NPK nutrients and also improved the plant panicles, number of tillers, 1000 grain weight, paddy yield, and plant height while decreasing concentration of Ni in grains [121]. The seeds’ priming by kinetin overcame the adverse effects of Cd by enhancing availability of soluble sugar in the endosperm, potentially aiding in seed germination and early seedling growth [122]. Exposure to 25 µM Hg resulted in the downregulation of genes involved in cytokinin signaling (*OsRR1*, *OsRR13*, *OsRR1 4*, *OsRR16*, and *OsRR111*) during long-term exposure, while genes associated with ethylene synthesis (*OsACS2*, *OsACO1*, *OsACO2*, *OsACO5*, and *OsACO6*) were significantly upregulated during short-term exposure [123]. This indicates a dynamic interaction between ethylene and CK signaling pathways in response to heavy metal stress. However, interaction of CK signaling with ethylene synthesis should be further explored to provide a more comprehensive understanding of the plant’s overall stress response. Similarly, treatment with trans-zeatin (ZT3) solutions at concentrations ranging from 20 to 40 μmol L^−1^ resulted in increased plant height, stem width, and the content of various metabolites such as 1-pyrroline, methylglyoxal, proline, and P5C. It also upregulated the expression of genes related to proline metabolism (*ProDH*, *P5CS2*, and *DAO4*) [120]. These studies demonstrate the potential of cytokinin treatments in alleviating the toxic effects of heavy metals and enhancing plant growth and stress tolerance in rice. Meanwhile, CK works as a positive regulator for IAA formation, and there is an endogenous regulatory homeostatic feedback loop that maintains optimal CTK and IAA levels for root and shoot development [124]. Under stress conditions, exogenous application of ABA significantly reduces isopentenyltransferases (IPTs) activity, a critical biosynthetic gene in CKs pathway, and upregulates oxidases/dehydrogenases [125]. However, future research should focus on a deeper understanding of CK signaling pathways in response to heavy metal stress. This includes identifying key regulatory elements and exploring potential targets for genetic manipulation to enhance stress tolerance. Moreover, field-level experiments should be conducted to explore the efficacy of CK treatments in real-world agricultural settings under different heavy metal concentrations, soil types, and environmental conditions.

### 10.1. Transcription Factors and Genes Regulated by Hormones in Rice under HM Stress

During Cd stress, the cell division and elongation are affected by ABA to regulate the growth of the elongation zone, indicating that the growth of rice roots under Cd-induced stress can be positively regulated by an appropriate level of ABA. The ABA biosynthesis pathway’s rate-limiting step is catalyzed by the 9-cisepoxycarotenoid dioxygenase (NCED) [126]. Hwang et al., 2010 reported that plants treated with ABA remarkably increased gene expression of *OsNCED* [127]. Zhao et al. reported that the Cd+TS and Cd+ABA differentially regulated the gene expression of *OsNCED1* and *OsNCED3*, indicating that the developmental stage of rice seedlings and ABA level under Cd-induced stress have a close relation with the regulation of ABA biosynthetic genes [19]. The catabolism and synthesis of ABA regulates its endogenous accumulation [126]. Zhao et al. reported that the concentration of ABA was higher in the first week and was lower in the second week during the growth of root system under Cd-stress, indicating that the ABA accumulation inhibits its own biosynthesis [19]. The ABA can partially alleviate the Cd caused growth inhibition. Zhao et al., 2014 also proposed that ABA signaling could be functioning through auxin signaling modification, affecting the signal transduction of MAPK and controlling the progression of the cell cycle [19]. During Cd stress, the signal transduction of ABA may occur through the following pathways. This indicates that ABA plays a vital role in regulating rice root growth under HMs stress in rice. The ABA biosynthesis pathway, particularly the NCED enzyme, is a major factor in this process. Studies showed that ABA levels are finely tuned in response to HM stress in rice, with feedback mechanisms at play. ABA partially mitigates HM-induced growth reduction, indicating its potential in stress mitigation. Furthermore, ABA’s interaction with auxin and MAPK signaling pathways further underscores its complex role in rice under HM stress response. Overall, understanding ABA’s involvement provides insights for developing novel strategies to enhance stress tolerance in rice facing HM stress.

### 10.2. The First Pathway: Altered Auxin Signaling via ABA Signal Transduction in Rice Plants Exposed to Cd Stress

Signaling of auxin is related to both Cd and ABA in rice. Signal transduction of ABA is altered through auxin under Cd stress [128,129,130]. During Cd stress response, ABA signaling is influenced by auxin distribution. In plant roots, auxin distribution is affected by both Cd and ABA treatments [131]. In Cd-stressed plants, Zhao et al., 2014 confirmed that the auxin distribution is influenced by the ABA concentration through GUS activity in 7-day-old Cd+ ABA/TS treated primary roots. During Cd stress, the MG132 and DRB detection revealed that the auxin distribution is regulated by ABA through protein degradation pathways [19].

Auxin transport is influenced by ABA signaling. Auxin signaling/transport is modulated by Cd and ABA [129]. Zhao et al., 2014 confirmed the auxin distribution regulated by ABA, using BFA protein transport inhibitor and TIBA polar transport inhibitor of auxin. TS+ Cd treatment affect the transport of polar auxin and retards root growth. Polar auxin transport is controlled by the efflux carriers called *OsPINs* [132]. Zhao et al., 2014 observed different polar auxin transport patterns between Cd+TS and Cd+ABA treatment groups, which could be due to *OsPIN* genes’ differential expression. In rice seedlings, the *OsPIN* genes, for instance, *OsPIN10b*, were differently regulated in Cd+TS and Cd+ABA treatment groups at both 7 and 11 days [19].

Auxin biosynthesis is regulated by ABA signaling in rice under normal conditions [133], as ABA treatment is reported for the induction of auxin-related biosynthesis genes and accumulation of IAA in rice roots for employing auxin as downstream signal for modification of radial expansion and root elongation [133]. During auxin biosynthesis, key roles are played by *OsYUCCA* gene family in rice and other plants by catalyzing the *N*-oxygenation of tryptamine to make *N*-hydroxytryptamine, which is a rate-limiting step in many plants’ biosynthesis of auxin [134]. In rice, *OsYUCCA2* gene expression is affected by both ABA and Cd [19,130]. Cd+TS (tungstate) and Cd+ABA differed in the *OsYUCCAs* expression patterns. The Cd+ABA-treated group resulted in lower IAA concentration compared to the Cd+TS-treated group, confirming that auxin biosynthesis is regulated by ABA signaling. Different endogenous and environmental signals can participate in triggering the auxin distribution and can affect the auxin transport and local auxin biosynthesis [135]. Modified gene expression of *OsPINs* and *OsYUCCAs* may contribute to the different auxin distribution patterns between Cd+TS and Cd+ABA treatment groups.

Moreover, ABA treatment is reported for inducing the *Auxin Response Factor2 (ARF2)* expression [136], while the expression of *AXR3/IAA17* and *AXR2/IAA7* are significantly repressed by ABA treatment [136]. The Cd- or ABA treated rice roots revealed down- or upregulation of *OsIAA* or *OsARF* [137]. Zhao et al., 2014 reported that ten *OsIAAs* and six *OsARFs*, e.g., *OsIAA17* and *OsARF1* auxin-responsive genes, respectively, were down- or upregulated by ABA + Cd. During different developmental stages of rice, ABA signaling pathway might function in different ways during Cd stress because most of the ABA/TS+Cd-regulated auxin genes were reported to have differentially regulated from 7 to 10 days. ABA partially counteracted the Cd-caused over-accumulation [19]. Zhao et al., 2014 confirmed that in roots under Cd-stress, ABA and auxin signaling interact while ABA acts upstream in the signaling pathways of auxin. Under Cd-stress response, auxin homeostasis may be determined by a certain ABA threshold level which alters auxin distribution and gene expression [19].

### 10.3. The Second Pathway: Cd-Stressed Rice Plants Regulate Cell Cycle by ABA Signaling

ABA or Cd treatment modifies the expression of various genes involved in cell cycles [138]. Under Cd stress, cell division and elongation is affected by ABA which regulates the growth of elongation zone in primary roots. Cd+ABA/TS treatment either downregulates or upregulates cell cycle genes such as *Oryza; CDKC;1*, *Oryza; CycD4;1*, *Oryza; CDKF;4* and *Oryza*; *CycT1;6*), suggesting that a part of cell cycle progression is controlled by ABA in plants under Cd stress. In plants under Cd stress, particular proteins involved in development and cell cycle machinery may be primarily targeted by ABA. ABA affects auxin signaling, which may lead to changes in cell-cycle related genes (*CDK*s and *CYC*s) expression. During Cd stress response, ABA has the potential to coordinate the cell cycle and auxin signaling [19]. The influence of ABA on auxin signaling pathways suggests a potential mechanism for altering the expression of cell cycle-related genes. This indicates ABA’s dual role in coordinating both cell cycle and auxin signaling in response to HM stress. Overall, these findings emphasize the intricate interplay between ABA, HM stress, and cell cycle regulation. Understanding these interactions provides comprehensive insights into potential strategies for augmenting stress tolerance in plants under HM contamination in rice.

### 10.4. The Third Pathway: MAPK Cascades Play a Role in ABA and Cd Signaling

Altered MAPK signaling is involved in ABA signal transduction of rice plants under Cd-stress. Treatment with Cd or ABA can lead to modified transcription of MAPK genes [137,139,140]. The regulation of MAPK genes by Cd+ABA differed from Cd+TS at 7 and 11 days, indicating that MAPK gene expression is linked to ABA levels. These genes play a key role in plant growth control, cell cycle progression, and auxin signaling [141]. In rice, ABA, MAPKs, and Cd control root growth by impacting auxin signaling and gene expression related to the cell cycle [137]. The MAPK signal transduction pathways may link ABA perception to root development and cell division through auxin signaling in response to Cd stress. The interactions of ABA with auxin signaling and MAPK, as well as the cell cycle, in addition to the target-specific-genes in Cd-stressed rice at certain developmental stages are well established [130,137,142]. Maintaining ABA homeostasis is crucial for the growth of rice roots under Cd stress, but some genes related to auxin, ABA, and the cell cycle may be regulated by other factors such as MAPKs, H_2_O_2_, and O_2_^•─^ [130,137,142]. The interplay between ABA, MAPKs, and Cd in rice significantly influences root growth by impacting auxin signaling and gene expression related to the cell cycle. This suggests that MAPK signal transduction pathways act as a bridge connecting ABA perception to root development and cell division in response to Cd stress. While maintaining ABA homeostasis is crucial for rice root growth under Cd stress, it is vital to acknowledge that certain genes related to auxin, ABA, and the cell cycle may also be influenced by other factors such as MAPKs, H_2_O_2_, and generation of superoxide anion radicals. 

## 11. Conclusions

Heavy metals usually decrease the endogenous level of auxins and cytokinin in rice. Exogenous application of auxins and their precursors could enhance the tolerance of rice to HM toxicity by overexpression of genes related to auxin biosynthesis. Exogenous application of cytokinin can also overcome the adverse effects of heavy metals in the early stages of development and can improve the uptake of nutrients. On the other hand, the endogenous GA level increases during HM stress while the exogenous application of GA alleviates HM toxicity by reduction of NO accumulation, oxidative stress, and Cd translocation. SA helps the plant to cope with HM stress by promoting the antioxidant compounds stimulation through interacting with other plant hormones such as gibberellin, abscisic acid, and auxins. The exogenous application of SA ameliorates the HM-induced increase of MDA, injury index, and electrolysis leakage. Membrane deterioration was also alleviated by SA application. The exogenous application of BRs and EBRs reduces the uptake, accumulation, and translocation of heavy metals by altering the antioxidant enzyme activities for ROS detoxification through upregulation of genes related to antioxidant enzymes, enhancing the metabolic activities, reduction of H_2_O_2_ production, and MDA. SLs (GR24) application reduces the uptake and accumulation of HMs in rice roots, enhances photosynthetic pigment contents, helps in the detoxification mechanisms of the cell, and stimulates plant growth. During heavy metal toxicity, the endogenous ABA level is increased in rice plants, partially due to the upregulation of ABA biosynthesis genes, indicating its involvement in defensive mechanisms against HM stress. The application of rice cultures with ABA increases the HM toxicity and inhibits the growth of young leaves. ABA does not affect Cd absorption but reduces the accumulation of Cd in rice leaves. The crosstalk between ABA, auxin, the cell cycle, and MAPK signaling pathways contributes to the regulation of gene expression and root development in rice plants under Cd stress. Phytohormones appear to function as signaling molecules and interact with redox signaling during metal exposure. This can trigger the expression of genes involved in plant defense systems, hence, cross-linked ROS and hormonal signaling systems Pb to a plant’s improved resilience to HM stress.

Understanding the intricate interactions between transcription factors, genes regulated by hormones, and signaling pathways provides valuable insights into the molecular mechanisms underlying stress responses in plants and can aid in the development of strategies to enhance plant tolerance to HM stress. Moreover, it is important to explore the role of multiple-hormone application simultaneously for deciphering the underlying molecular mechanism and their subsequent effects on endogenous phytohormones level. It is necessary to explore the effective phytohormone content of rice species to build a cost-efficient and eco-friendly crop management strategy. Nevertheless, phytohormones and the signaling molecule system are complicated; thus, phytohormone analysis should be conducted together with other analyses of physiological and biochemical attributes of rice to gain better insights into phytohormone-induced untargeted metabolism as well as the alteration of associated microbial biota of rice under HM toxicity. 

## Figures and Tables

**Table 1:** The effect of auxin application on rice during heavy metal stress.

Trearment	Application	Conc.	Effect	References
Cd (30 mg kg^−1^ of soil)	Exogenous Se and L-TRP (auxin precursor)	10^−5^ M	Enhanced growth and yield under Cd stress by stabilizing endogenous auxin levels and decreasing Cd translocation to rice grains	[21]
As	Exogenous IAA	3.0 M	Reduced As-induced stress more efficiently when used in combination with selenium and improved chlorophyll content, proline, and cysteine; lowered protein content inhibition and DNA damage; reduced lipid peroxidation	[22]
-	Exogenous indole-3-butyric acid (IBA)	1 µM	Increased synthesis of nitric oxide in lateral roots and heme oxygenase activity	[23]
Mercury (60 µM)	Exogenous nitroprusside (SNP or NO)	200 µM	Induced auxin transport in roots and improved resistance to Hg-stress, decreased Hg uptake and transportation in roots and shoots, decreased auxin levels during iron deficiency. Antioxidant activity was not enhanced by SNP	[24]
Cd/As	Exogenous IAA or IBA	100 mM	Mitigated alterations in root system caused by Cd stress by increasing nitric oxide content and lateral root production and *AUX1* expression	[25]
Hexavalent chromium (300 µM)	OsMYB-R1 overexpressing lines	-	Rice plants showed higher auxin accumulation, overexpression of *OsMYB-R1*-induced antioxidative genes such as *CAT*, *SOD*, guaiacol peroxidase; and regulated salicylic acid signaling under Cr 300 µmol L^−1^ and other abiotic stresses	[26]
CdSO4 and Na2HAsO4 (100 µmol L^−1^)	IAA or IBA	10 µM	Altered *OsAUX1*, *OzYUCCA1*, *OsASA2* IAA-biosynthesis gene and *OsYUCCA1* expression was downregulated under As and Cd toxicity, while OsASA2 expression was not influenced with or without Cd and As. 100 µmol L^−1^ CdSO4 and 100 µmol L^−1^ Na2HAsO4·7H_2_O	[25]
As	IAA	2.0 μmol L^−1^	Enhanced grain biomass, reduced As translocation, decreased the As concentration in rice grains	[27]
Cd	NAA	1.0 and 10 μmol L^−1^	Enhanced plant biomass, restricted seedling growth in both wild type and transgenic *pmei12* lines	[20]
Vanadium (0–2 mM)	-	-	Five genes encoding auxin response transcription factors (*OsIAA*) as well as enhanced abscisic acid (ABA) and jasmonic acid (JA) expression in hormone signaling pathways. Upregulated ATP-dependent GSH-conjugated transport, ATP binding cassette (ABC) transporter, and markedly reduced the expression of divalent cation transporters, drug/metabolite transporter (DMT), and zinc/iron permease (ZIP)	[28]

**Table 2:** The effect of exogenous application of abscisic acid in rice during Heavy metal stress.

Treatment	Application	Conc.	Effects/Mechanism	References
CuSO_4_ and CdCl_2_	ABA	10 μM	Upregulation of stress membrane protein (*OsSMP1*), increasing tolerance to heavy metal stresses but increasing sensitivity to ABA	[36]
CdCl_2_	Pretreatment with ABA	10 μM	Reduced transpiration rate, decreased Cd content, and enhanced Cd tolerance of TN1 seedlings. ABA content enhanced in roots and leaves of Cd-tolerant cultivar	[37]
Cd	ABA	100 μM	Stomatal closure, reduced transpiration rate, and dramatically reduced the accumulation of Cd in the leaves	[32]
Pb, Cd, As	ABA	-	Increase in ABA concentrations; signaling and biosynthesis genes of ABA are upregulated	[17]
Cd, Cu	-Si	-	Increased level of endogenous ABA in Si-plants after 10 days, triggered heavy metal transporters (*OsHMA2* and *OsHMA3*) genes	[34]
Cd	ABA	-	Upregulation of ABA biosynthesis genes, positive correlation between Cd tolerance and endogenous ABA content, rapid ABA production detected in roots and leaves of Cd-tolerant rice genotypes	[37]
Cd (0.1 mM) or Nickel (0.5 mM)	ABA	19 µM	Potentiate the effects of heavy metals by accumulating carbohydrates and inhibiting growth	[35]
As(III) (25 and 50 mM L^−1^)	pretreated with ABA	10 µM	As altered phosphatase, H^+^/ATPase, alkaline phosphatase, ROS, antioxidative and proline biosynthesis genes and ABA regulated phosphatase, H^+^/ATPase and alkaline phosphatase to moderate phosphate and upregulated antioxidative biosynthesis genes and downregulated ROS biosynthesis genes	[40]
As(V) (25 µM)	-	-	Two ABA biosynthesis genes, *OsNCED2* and *OsNCED3*, were strongly increased and 4 ABA signaling genes were upregulated. Expression of GARP-G2-like and C3H transcription factors was specifically modulated by As(V) stress. MAPKs activity was enhanced.	[33]
Pb (0.25 mM Pb)	Pretreatment with ABA	0.1 g m^−3^	Restricted amount of Pb translocated from roots to shoots, decreased malondialdehyde and H_2_O_2_ contents in leaves, and alleviated Pb-induced decrease in plant growth and leaf chlorophyll content and improved increased ascorbate peroxidase and catalase activities	[42]
Vanadium (1 mM)	-	-	Expression of ABA hormone signaling pathways increased. NAC (NAM, ATAF, CUC) proved to be V-specific transcription factor	[28]

**Table 3:** The effect of ethylene application on heavy metals in rice.

Treatment	Application	Effect	References
Cr	Ethylene	Increased expression of four ethylene biosynthesis-related genes (*ACS1*, *ACS2*, *ACO4*, and *ACO5*)	[21]
200 μM Cu^2+^	Ethylenediamine-N, N′-disuccinic acid (400 μM)	Enhance plant tolerance potential to excess Cu toxicity through alleviating Cu-induced poisonous effects. Modulated the mRNA level of Cytochrome P450 gene, *OsHMA9*, and sulfate transporter gene	[49]
Copper oxide nanoparticles (CuO-NP) (450 mg L^−1^)	Ethylene biosynthesis and signaling antagonists cobalt and silver	Reduces the extent of ultrastructural and stomatal damage by controlling ROS accumulation in rice seedlings and cellular ultrastructural damages	[44]
ZnO NPs	-	Upregulation of *ACS2* and *ACS6* transcripts responsible for ethylene biosynthesis	[47]
Mercury and/or Se	Selenium (Se)	Triggered the ethylene transduction gene in *Oryza sativa* and regulated the synthesis of ethylene and osmotic balance	[48]
Hexavalent chromium		Overexpressed the *ACO5*, *ACO4*, *ACS2,* and *ACS1* genes to enhance ethylene biosynthesis to regulate Cr-induced oxidative stress	[5]
Chromium	-	Modulation of ethylene biosynthesis and signaling, vesicle trafficking, and ROS level	[46]
Chromium	-	Upregulation of ethylene biosynthesis *AP2*/*ERF* gene family	[15]
As (25 µM)	-	APETALA2/ethylene response factor expression was increased. Two ethylene biosynthesis genes, *OsACS2* and *OsACO4*, were strongly increased, and three ethylene signaling genes were upregulated.	[33]

**Table 4:** The effect of SLs on heavy metals in rice.

Treatment	Application	Effect	References
As	SL deficient rice	Severe growth abnormalities in trigolactone-deficient mutants while wild type showed reduced As uptake and accumulation and reduced phosphate-transporters encoding gene expression, enhanced transcript accumulation of *CAT*, *SOD* and *APX* genes and lowered expression of phosphate tranporter genes.	[55]
Cd or As	GR24	Effectively inhibited Cd or As uptake by rice plants but Cd accumulation and translocation from root to shoot was not decreased.	[58]
-	SLs	Play an active role in structuring rhizomicrobiome and mediation of distinct metabolic pathway.	[53]
Phosphate deficiency	Exogenously applied GR24 (1 µM)	GR24 restored normal leaf senescence in SL-deficient mutants.	[54]

**Table 5:** The effect of JA on heavy metals in rice.

Treatement	Application	Concentration	Effects	References
As (III)	Exogenous MeJA	0.25 μM	Enhanced biomass and chlorophyll content and increased antioxidant enzyme activities, decreased accumulation of total AsIII content (root + shoot) and modulated JA signaling pathway genes downstream (*OsCOI*, *OsJAZ3*, *OsMYC2*)	[68]
Cd (50 μM)	methyl jasmonate	5 μM	Enhanced Cd-tolerance and antioxidant response, lowered Cd uptake, an improved membrane integrity and ‘switching on’ of the JA-biosynthesis by lipoxygenase (LOX)	[81]
As (0, 25 and 50 µM)	MJ	(0, 0.5 and 1 µM)	Alleviated the negative effects of As toxicity and increased chlorophyll contents, biomass production, and Fe accumulation, decreased the oxidative stress by regulating ASA–GSH cycle and antioxidants. Reduced *Lsi1*, *Lsi2*, and *Lsi6* expression.	[65]
As(V) (2 μM)	Pretreatment with JA	0.5 to 5 µM	Decreased the As concentrations in the roots and shoots, with the effect being significant for shoot As concentration	[69]
Pb (150 and 300 µmol L^−1^)	MJ	0.5 µM or 1 µM	Altered *HMAs*, *ABCC1*, *PCS1* biosynthesis genes, regulated antioxidant and proline, glyoxalase system, and phytochelatins. Reduced MDA and H_2_O_2._ Immobilized Pb in root and reduced accmulation in shoot.	[80]
As (50 μmol L^−1^)	MJ	0.5 and 1 µM	Altered *ABCC1*, *GSH1*, PCS genes, antioxidative biosynthesis genes, glyoxalase regulated genes and MJ regulated the expression of *ABCC1*, *HMAs*, *PCS1,* and two genes for As sequestration. MJ also upregulated antioxidant and proline biosynthesis genes and downregulated MDA, MG, and H_2_O_2_ expression	[65]
Se(IV) (25 μM) (Na2SeO3)	MeJA	0.1–1.0 μM	Altered *OsSBP1*, *OsNIP2;1*, *OsPT2,* and low concentration of MJ depressed the gene expression of *OsPt2* and *OsNIP2;1* in roots and *OsSBP1*, *OsCS*, *OsNIP2;1,* and *OsPT2* in shoot to hinder Se uptake	[56]
Aluminium (0.5 and 1 mM L^−1^)	MJ	0.5 and 1 µM	Altered *ABCC1*, *GSH1*, and *PCS* and MJ upregulated the expression of *ABCC1*, *GSH1*, and *PCS* for Al sequestration in the vacuole	[79]
As (III) (25 mM L^−1^)	MJ	0.5 and 1 µM	Improved chlorophyll metabolism, phytochelatins, and glutathione. Altered *OsCOI*, *OsMYC2*, *OsJAZ3*, *OsINT5*, *OsLsi6*, *OsNIP3;1*, *OsLsi1;2*, *OsNIP1;1*, *OsABCC2*, *OsNRAMP1*, and *OsPCS2* and MJ treatment downregulated AsIII absorption (*OsNIP1;1 OsNIP1;3*, *OsLsi1 and OsLsi2*), translocation (*OsINT5* and *OsLsi6*) and detoxification (*OsABCC2*, *OsNRAMP1*, and *OsPCS2*) genes to cope up As toxicity	[68]
As(V) (25 uM)	-	-	Five JA biosynthesis genes (*OsDAD1;2*, *OsLOX2;1*, *OsAOS2*, *OaAIM1 and OsJAR1;2*) and six JA signaling genes were up regulated	[33]
Antimony (10 to 50 mg L^−1^)	-	-	Significantly increased methyl jasmonate in rice roots for reducing the toxic effects	[66]
Vanadium (1 mM)	-	-	Jasmonate ZIM domain family was upregulated and expression of jasmonic acid hormone signaling pathways (6 genes) and biosynthesis (3 genes) increased	[28]

**Table 6:** The effect of BRs on heavy metals in rice.

Treatment	Application	Concentration	Effects	References
As & Cd	Exogenous Br24 and Br28	0.2 or 0.02 μM	Decreased Cd and As accumulation and translocation to the rice grains.	[68]
Cd and As	Iron plaque (IP) and Br	20 or 60 mg Fe^2+^ dm^−3^	Impedes accumulation and transports Cd and As.	[96]
Iron (250 and 6250 μM)	24-epibrassinolide (EBR)	10 nM	Increased ROS scavenging, enhanced the activities of enzymes such as peroxidase, ascorbate peroxidase, catalase, and superoxide dismutase, modulated arenchyma area for reducing Fe mobilization in root.	[98]
Chromium (100 µM)	Seed Priming with Brassinosteroids (EBL)	0.01 µM	Altered antioxidative defense-associated genes and upregulated *CAT*, *APX*, and *POD*, while downregulating the *MDA*, *H2O2*, *SOD*, EL, and mitigated sub-cellular damages to ameliorate Cr toxicity.	[90]
Aluminum (400 μmol L^−1^)	Seed priming with 24-epibrassinolide	(0.01 μM)	Altered *APX08*, *CATa*, *CATb*, *APX02*, *SOD*-Fe2, and *SOD*-C*u*-Zn and upregulated BRs and antioxidant defensive genes.	[99]
As (40 mg kg^−1^) and Cd (5 mg kg^−1^)	Spray Br28 or Br24	10^−7^ mg	Altered antioxidative-related genes and increased Fe plague, which improved Mn, Cu, and Zn uptake in roots and restricted Cd and enhanced As root uptake and translocation.	[92]
Iron (250 and 6250 μmol L^−1^)	EBR	10 nM	Altered antioxidative defense-related genes and decreased ROS, and increased carboxylation, CAT, SOD, and POD activities.	[98]
As 5 µM	2,4-epibrassinolide (EBL)	0.2 µM	EBL significantly increased the content of carotenoid by 5.8% and significantly decreased As content in the roots by 32.5%.	[93]
Cd (20 mol L^−1^)	Foliar Spraying of brassinolide	0.1 mM	Increased root length and root surface area, and CAT, SOD, and POD activities were significantly improved and decreased the Cd content of rice by transforming Cd into immobile forms and fixing in the cell wall.	[95]

**Table 7:** The effect of SA on heavy metals in rice.

Treatment	Application	Concentration	Effects/Mechanism	References
HgCl_2_ (10 µM) or PbCl_2_ (10 µM)	Seed germinated on SA moistened paper discs.	100 µM	Alleviated the membrane deterioration caused by lipoxygenase (LOX). Reduced MDA and enhanced H_2_O_2_ under Pb stress	[108]
Cd (25 μM)	SA	100 μM	Showed elevated photosynthetic pigment content, on-protein thiol content, relieved the growth inhibition, and lowered the ROS accumulation. Upregulated *OsHMA3* and *OsPCS1* and lowered *OsNRAMP2* expression	[110]
Cd (2.5 μM)	Foliar Spray of SA	0.1 mM	Increased the leaf’s Cd content at mature stage and decreased the accumulation of Cd in grains by depositing and fixing in cell wall of leaves	[111]
CdCl_2_ (50 μmol L^−1^)	Pretreatment of rice roots with SA	10 μM	Improves root growth; reduces ROS level, and membrane damage; enhances SOD, POD and CAT activities as well as GSH, and AsA contents; improves non-protein thiols’ concentration	[107]
As (V) (25 and 50 μM)	SA	10 μM	Plant growth and As(V) induced oxidative stress while drastically reducing the roots to shoot translocation of AsV. *OsNRAMP5* and *OsFRDL1* were enhanced	[112]
Chromium (100 mmol L^−1^)	SA	100 μM	Alters *OsPCS1*, *OsMT1* and *OsHMA3* and plant response SR, CAT, POD, and SOD increased to regulate Cr-mediated ROS in rice seedlings	[110]
As 10 μmol L⁻¹	SA	(2.0 μmol L^−1^)	Increased root and shoot elongation, biomass, total root length, root surface area, root volume, and root tip number	[27]
Pb (0.05, 0.15 and 0.25 mmol L^−1^)	SA pretreatment	0.1 mmol	Increases seedling shoot, and root length; improves chlorophyll content; reduces peroxide levels; alters SOD and APX activities of hybrid rice cultivar	[105]
Cr+6	OsMYB-R1 overexpressing rice	-	Controls the crosstalk of auxin and salicylic acid signaling and other genes in response to Cr stress	[26]
Cr(VI)	SA application in solution culture	100 μM	Three genes (*OsPCS1*, *OsMT1*, and *OsHMA3*) involved with vacuolar sequestration showed significant upregulation due to SA treatment. Modulated salicylic acid signaling molecule calcium-dependent protein kinases, to activate the stress-responsive downstream genes (Peroxidases, Glutathione S-transferases, Osmotins, Heat Shock Proteins, Pathogenesis Related-Proteins)	[106]

**Table 8:** The effect of GA on heavy metals in rice.

Treatment	Application	Conc.	Effects	References
-	GA3	1.0 and 10 (μmol L^−1^)	Higher growth rate and seedling height in both wild type and *pmei12* lines compared to the control	[20]
Cd (0.1 mM) or Ni (0.5mM)	Exogenous GA3 application	14 μM	Partially reversed the effects of heavy metals and stimulated growth. Activated mobilization of carbohydrates in seeds	[35]
Cd or Nickel treatment on rice plants	Exogenous GA_3_	1.4 × l0^−5^ M	Adverse effects of the metals on the nutrient acquisition were not alleviated and decreased Ca content in Ni-plants. Endogenous GA reduced under stress.	[35]
Fe and Mn plaque	Exogenous (GA_3_) application through spraying	0.18 mM GA_3_	Decreased Fe plaque, but increased Mn plaque	[116]
As (10 μmol L⁻¹)	Seedlings were pretreated GA	(2 μmol L^−1^)	Shoot biomass and root elongation significantly increased. As concentration was reduced.	[27]
Cd	Exogenous GA	-	Decreased the fixation of Cd in the root cell via lowering hemicellulose content, decreased the expression of *OsNRAMP5* and *OsCd1*, increased *OsHMA3* and *OsCAL1* and accelerated cell wall Cd exclusion mechanism. Lowered endogenous NO production and antioxdant enzymes.	[114]
Cd (20 mol L^−1^)	Foliar Spraying of Gibberellins	0.1 mM	Root length and root surface area, and CAT, SOD, and POD activities were significantly improved; decreased the Cd content of rice by transforming Cd into immobile forms and fixing in the cell wall.	[95]

**Table 9:** The effect of cytokinin on heavy metals in rice.

Treatment	Application	Conc.	Effects	References
Nickel sulfate (130 mg kg^−1^)	Rice seedlings dipped in kinetin solution	10^−4^ M	Improved the plant panicles, number of tillers, 1000 grain weight, paddy yield, and plant height while decreasing concentration of Ni in grains and enhanced in in shoot. NPK uptake improved.	[121]
CdCl_2_ (4, 12 & 30 ppm)	seeds Priming with Mg (NO_3_)_2_ and kinetin	5 ppm	Overcome the adverse effects/phytotoxicity of the HMs by improving α-amylase activity and enhancing availability of soluble sugar in the endosperm	[122]
Mercury (25 µM)	-	-	Genes involved in cytokinin signaling (*OsRR1*, *OsRR13*, *OsRR14*, *OsRR16,* and *OsRR111*) were downregulated in long-term Hg exposure and 5 ethylene (ET) synthesis genes–*OsACS2*, *OsACO1*, *OsACO2*, *OsACO5* and *OsACO6*– were significantly increased in short-term Hg exposure. Activated calcium accmulation and mitogen-activated protein kinase (MAPK).	[123]
As(V) (25 µM)	-	-	Two CK—inactivation genes (*OsCKX4* and *OsCKX5*) were strongly increased, 1 CK biosynthesis gene (*OsIPT4*) was decreased, and 2 CK signaling genes (*OsHKL1/OsCRL4*) and (*OsRR20*), were downregulated	[33]
-	Trans-zeatin (ZT3) solutions treatments	20 to 40 μmol L^− 1^	Increased plant height and stem width, 1-pyrroline, methylglyoxal, proline, and P5CS and OAT activities, enhanced P5CS and OAT activities, and reduced glutamic acid contents. *ProDH*, *P5CS2*, and *DAO4* expression upregulated.	[120]

## Supplementary Materials

The following supporting information can be downloaded at: https://www.mdpi.com/article/10.3390/metabo13101036/s1, Table S1: List of Abbreviations.
