# Supplementary material for: Novel Insights into Exogenous Phytohormones: Central Regulators in the Modulation of Physiological, Biochemical, and Molecular Responses in Rice under Metal(loid) Stress
Source: Metabolites. 2023 Sep 26;13(10):1036. doi: 10.3390/metabo13101036 (PMC10608868; doi:10.3390/metabo13101036)
Supplement: Supplementary file 1 [file metabolites-13-01036-s001.zip › metabolites-2597747-supplementary.pdf]

Table S1. List of Abbreviations

Abbreviation	Term
ABA	Abscisic Acid
NCED	9-cis-Epoxycarotenoid Dioxygenase
OsNCED	Oryza sativa 9-cis-Epoxycarotenoid Dioxygenase
Cd	Cadmium
TS	Tungstate
IAA	Indole-3-Acetic Acid (Auxin)
GUS	β-Glucuronidase
BFA	Brefeldin A
TIBA	2,3,5-Triiodobenzoic Acid
OsPINs	Oryza sativa PIN-FORMED proteins
OsYUCCA	Oryza sativa YUCCA
ARF2	Auxin Response Factor 2
AXR3/IAA17	Auxin Response 3/Indole-3-Acetic Acid 17
AXR2/IAA7	Auxin Response 2/Indole-3-Acetic Acid 7
CDKC;1	Cyclin-Dependent Kinase C-1
CycD4;1	Cyclin D4;1
CDKF;4	Cyclin-Dependent Kinase F-4
CycT1;6	Cyclin T1;6
MAPK	Mitogen-Activated Protein Kinase
H2O2	Hydrogen Peroxide
O2—	Superoxide Anion Radical
NO	Nitric Oxide
ROS	Reactive Oxygen Species
SLs	Strigolactones
BRs	Brassinosteroids
EBRs	24-Epibrassinolide
HM	Heavy Metal

SA	Salicylic Acid
Cd	Cadmium
MDA	Malondialdehyde
H2O2	Hydrogen Peroxide
AsV	Arsenate
GA	Gibberellic Acid
Fe	Iron
Mn	Manganese
Ni	Nickel
CK	Cytokinin
P5C	Pyrroline-5-Carboxylate
ProDH	Proline Dehydrogenase
P5CS2	Pyrroline-5-Carboxylate Synthetase 2
DAO4	Diamine Oxidase 4
OAT	Ornithine Aminotransferase
NO	Nitric Oxide
OsCKX5	Oryza sativa Cytokinin Oxidase/Dehydrogenase 5
OsCKX4	Oryza sativa Cytokinin Oxidase/Dehydrogenase 4
OsIPT4	Oryza sativa Isopentenyltransferase 4
OsRR20	Oryza sativa Response Regulator 20
OsHKL1/OsCRL4	Oryza sativa Histidine Kinase-like 1 / Cytokinin Receptor-like 4
OsRR1	Oryza sativa Response Regulator 1
OsRR13	Oryza sativa Response Regulator 13
OsRR14	Oryza sativa Response Regulator 14
OsRR16	Oryza sativa Response Regulator 16
OsRR111	Oryza sativa Response Regulator 111
OsACS2	Oryza sativa 1-Aminocyclopropane-1-carboxylic Acid Synthase 2
OsACO1	Oryza sativa 1-Aminocyclopropane-1-carboxylic Acid Oxidase 1
OsACO2	Oryza sativa 1-Aminocyclopropane-1-carboxylic Acid Oxidase 2

OsACO5	Oryza sativa 1-Aminocyclopropane-1-carboxylic Acid Oxidase 5
OsACO6	Oryza sativa 1-Aminocyclopropane-1-carboxylic Acid Oxidase 6
ZT3	Trans-Zeatin
NPK	Nitrogen, Phosphorous, and Potassium (nutrients)
HMs	Heavy Metals
Cd	Cadmium
Pb	Lead
As	Arsenic
Zn	Zinc
OsHMPs	Oryza sativa Heavy metal-associated proteins
MAPK	Mitogen-Activated Protein Kinase
IAA	Indole-3-Acetic Acid
ARF	Auxin Response Factor
PIN	Pin-Formed
YUCCA	YUCCA (auxin biosynthesis gene)
NAA	Naphthaleneacetic Acid
L-TRP	L-Tryptophan
IBA	Indole-3-Butyric Acid
Hg	Mercury
CAT	Catalase
SOD	Superoxide Dismutase
Cr	Chromium
OsAUX1	Oryza sativa AUX1 (auxin transporter)
OzYUCCA1	Oryza sativa YUCCA1 (auxin biosynthesis)
OsASA2	Oryza sativa ASAT2 (Amino acid synthesis)
CdSO4	Cadmium Sulfate
Na2HAsO4	Sodium Arsenate
OsIAA	Oryza sativa Indole-3-Acetic Acid
V	Vanadium

OsMYB-R1	Oryza sativa MYB-RELATED 1
YUC	YUCCA (auxin biosynthesis gene)
TAA1	Tryptophan Aminotransferase of Arabidopsis 1
WOX11	WUSCHEL-RELATED HOMEOBOX 11
OsNCED	Oryza sativa NINE-CIS-EPOXYCAROTENOID DIOXYGENASE
CuSO4	Copper Sulfate
CdCl2	Cadmium Chloride
ROS	Reactive Oxygen Species
g	Gram
m-3	Cubic Meter
H2O2	Hydrogen Peroxide
µM	Micromolar
OsSMP1	Oryza sativa SULFUR MOBILIZATION PROTEIN 1
Ni	Nickel
mM	Millimolar
mg	Milligram
DWARF gene	Gene associated with plant growth regulation
OsABCC1	Oryza sativa ATP-Binding Cassette C1
OsPCS1	Oryza sativa Phytochelatin Synthase 1
OsGSH2	Oryza sativa Glutathione Synthetase 2
Gr24	Strigolactone GR24
SLs	Strigolactones
JAs	Jasmonates
ABCG	ATP-Binding Cassette G (transport proteins)
HMAs	Heavy Metal ATPases
PCS1	Phytochelatin Synthase 1
MDA	Malondialdehyde
H2O2	Hydrogen Peroxide
MG	Methylglyoxal

MeJA	Methyl Jasmonate
Se+4	Selenium
OsSBP1	Oryza sativa Squamosa Promoter Binding Protein 1
OsNIP2;1	Oryza sativa NOD26-like intrinsic protein 2;1
OsPT2	Oryza sativa Phosphate Transporter 2
Al	Aluminium
OsCOI	Oryza sativa Coronatine Insensitive 1
OsMYC2	Oryza sativa MYC2
OsJAZ3	Oryza sativa Jasmonate ZIM-Domain Protein 3
OsINT5	Oryza sativa Inositol Transporter 5
OsLsi6	Oryza sativa Lsi6
OsNIP3;1	Oryza sativa NOD26-like intrinsic protein 3;1
OsLsi1;2	Oryza sativa Lsi1;2
OsNIP1;1	Oryza sativa NOD26-like intrinsic protein 1;1
OsABCC2	Oryza sativa ATP-Binding Cassette C2
OsNRAMP1	Oryza sativa Natural Resistance-Associated Macrophage Protein 1
OsPCS2	Oryza sativa Phytochelatin Synthase 2
Br	Brassinosteroids
BRs	Brassinosteroids
EBRs	Epibrassinolides
APX08	Ascorbate Peroxidase 08
APX02	Ascorbate Peroxidase 02
CATb	Catalase b
CATa	Catalase a
SOD-Fe	Superoxide Dismutase - Iron form
SOD-Cu-Zn	Superoxide Dismutase - Copper-Zinc form
